# Cancer risk in relation to body fat distribution, evaluated by DXA-scans, in postmenopausal women – the Prospective Epidemiological Risk Factor (PERF) study

**DOI:** 10.1038/s41598-019-41550-1

**Published:** 2019-03-29

**Authors:** Line Mærsk Staunstrup, Henning Bay Nielsen, Bente Klarlund Pedersen, Morten Karsdal, Joseph Patrick Michele Blair, Jesper Frank Christensen, Cecilie Liv Bager

**Affiliations:** 1 ProScion, Herlev, Denmark; 2 University of Copenhagen, Health, Copenhagen, Denmark; 3 The Centre of Inflammation and Metabolism and the Centre for Physical Activity Research, Rigshospitalet, University of Copenhagen, Copenhagen, Denmark; 4 Nordic Bioscience, Herlev, Denmark

## Abstract

Studies with direct measures of body fat distribution are required to explore the association between central and general obesity to cancer risk in postmenopausal women. This study investigates the association between central obesity and general obesity to overall/site-specific cancer risk in postmenopausal women. The analysis included 4,679 Danish postmenopausal women. Body fat distribution was evaluated by whole-body dual-energy X-ray absorptiometry scanners. Cancer diagnoses were extracted from the Danish Cancer Registry and multivariable Cox regression models explored the association between cancer risk and central obesity after adjusting for BMI. Our results showed that high central obese women had a 50% increased risk of overall cancer relative to low central obese women (Q1vs.Q4: [HR:1.50, CI:1.20–1.88]). For site-specific cancers, central obesity was significantly associated with Respiratory (Q1vs.Q4: [HR:2.01, CI:1.17–3.47]), Gastrointestinal (Q1vs.Q4: [HR:1.55, CI:0.99–2.41]) and Female genital organs (Q1vs.Q4: [HR:1.95, CI:1.00–3.78]) cancer diagnoses. Sub-analyses stratified by smoking-habits found a significant association between central obesity and a cancer diagnosis for current (Q1vs.Q4: [HR:1.93, CI:1.25–2.99]) and former smokers (Q1vs.Q4: [HR:1.90, CI:1.23–2.94]). These analyses suggest that central obesity is associated with some cancers in postmenopausal women independent of BMI.

## Introduction

Obesity is an increasing problem in developed countries comprising a major global health challenge^1^. A national health profile made by the Danish Health Authority showed that 51.5% of Danish women in the age range of 65–74 years were overweight or obese in 2017^2^. The same trend of increasing obesity among the elderly is observed in the rest of the western world^3^.

Several epidemiological studies have evaluated the link between obesity and cancer risk^4–6^. There is strong evidence that obesity, measured by BMI, is a risk factor for a variety of different cancers, including oesophagus, pancreas, liver, colorectum, breast (postmenopausal) and ovary cancer^6^. It is estimated that almost 10% of cancer incidences in 2012 were due to overweight or obesity^7^. However, the current body of evidence relies almost exclusively on indirect anthropometric measures of obesity, e.g. body mass index (BMI), waist circumference (WC) and/or waist-to-hip ratio (WHR)^8^, which are associated with major limitations. In women, menopause initiates a shift of body fat towards a higher level of abdominal/visceral adiposity^9^, which is not captured by BMI. Moreover, anthropometric measures fail to differentiate between lean and fat mass^10^. Thus, studies with direct measures of body fat distribution are required to explore the association between body fat distribution and cancer risk in postmenopausal women. To this end, dual-energy X-ray absorptiometry (DXA) scanning is the gold standard for direct determination of whole-body and regional distribution of lean and fat mass, but only a few large-scale epidemiological studies have applied this method^11^.

The present study explored the association between body fat distribution in postmenopausal women and cancer risk using data from the Prospective Epidemiological Risk Factor (PERF) study^12^.

Here, DXA scans were available at baseline and data for cancer diagnoses along with death records for 5,855 Danish postmenopausal women were retrieved over a 12-year follow-up period. Accordingly, we examined the association between obesity and overall cancer risk in the postmenopausal women included in the PERF cohort. Obesity was measured both by BMI and trunk-to-peripheral fat ratio, to reflect overall and central obesity respectively. The principal objective was to explore the potential discrepancy between whole-body and regional obesity derived from DXA-scans adjusted for known risk factors for cancer and mediators of obesity. We further explored the association between obesity and site-specific cancer risk, and finally stratified our analyses by smoking habits as a potential moderating factor^13^.

## Methods

### Study population

PERF is an observational, prospective cohort consisting of 5,855 Danish postmenopausal women^12^. Women who previously participated in clinical prevention trials or had been screened for inclusion in randomized clinical prevention trials at the Center for Clinical and Basic Research (CCBR) were invited to participate in PERF. The characteristics obtained in the PERF cohort including lifespan and disease history are comparable with known distributions from elderly Danish women according to statistics Denmark^14^. The study was designed to obtain knowledge about the determinants of age-related diseases in elderly women and the participants are considered representative of postmenopausal Danish women.

### Data collection and variable definition

The PERF baseline population was examined between 1999 and 2001 at CCBR located in either Ballerup or Aalborg (Denmark)^12^. The examination included a comprehensive medical workup along with an interview with a doctor or a nurse covering questions related to lifestyle, demographics, family disease history, reproduction, smoking history, alcohol consumption and level of education. All participants had their weight and height measured which allowed for calculation of BMI (kg/m^2^).

Participants of the study were matched to the Danish Cancer Registry, the National Danish Causes of Death Registry, the Danish National Patient Register^15^ and the Danish adult diabetes registry^16^ through a personal subject identification number (CPR-number). Cancer categories were classified according to WHOs international Classification of Diseases 10 (ICD10) [Non-solid and non-malignant tumours (D04, D10–48, C44, C81–96, N87); Breast cancers (C50, D05); Female genital organs (FGO) cancers (C51–58, D06); Respiratory cancers (C30–39, D02); Gastrointestinal (GI) cancers (C15–26, D00–01); and Other cancers (C00–14, C40–41, C43–49, C64–80, D03, D07, D09)]. Co-morbidities at baseline were extracted from the Danish National Patient Register and the Danish adult diabetes registry.

### Body fat measurements

Regional adiposity (legs, arms, trunk and head fat mass (g)) measures were performed with DXA scanners (HOLOGIC QDR-4500, HOLOGIC QDR-2000, Lunar Prodigy). Central obesity was defined as the trunk-to-peripheral fat ratio^17^ and was calculated by division of fat mass in the trunk area with fat mass in arms and legs. DXA-scans were normalized into z-scores for analysis with the largest population as the reference (HOLOGIC QDR-4500). Before doing so it was insured that the three groups had the same age, weight and height distribution. Central obesity was normally distributed and DXA-scan measures of this cohort were consistent with reference values provided by T. Kelly *et al*.^18^. For analysis and comparison, the central obesity and BMI variables were divided into quartiles.

### Inclusion criteria

**Figure 1:**
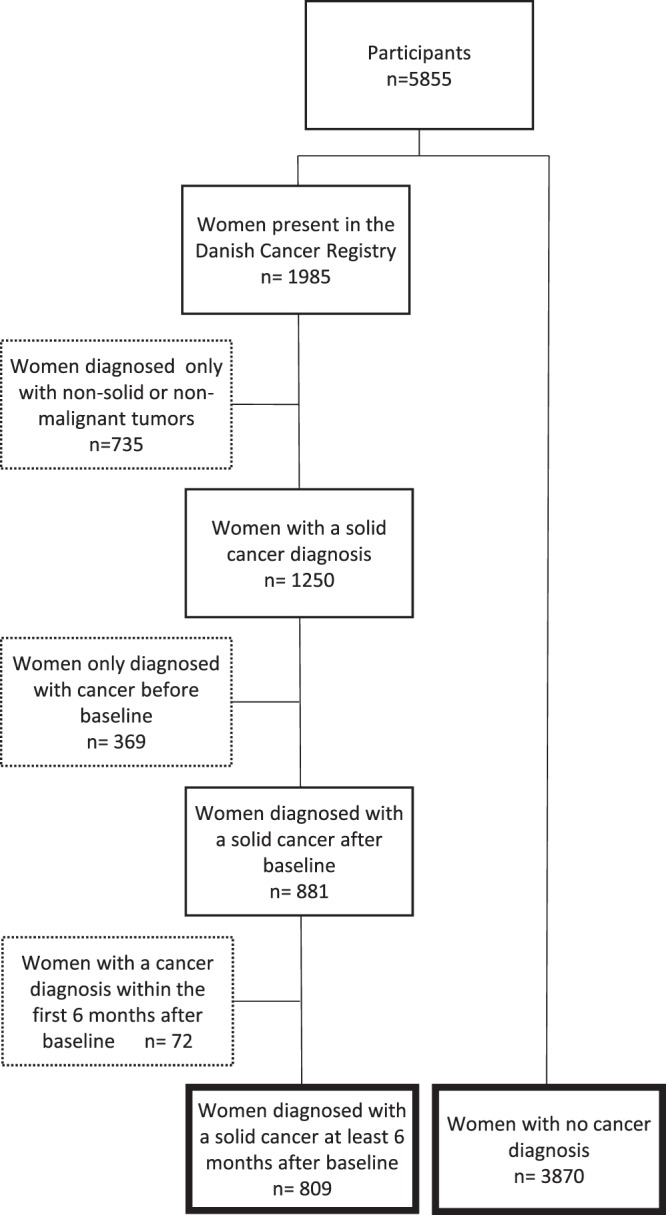
Flow chart showing the organization and categorization of postmenopausal women included in the study.

Women with a diagnosis of solid cancer after baseline and women not present in the Danish Cancer Registry (non-cases) were included in the analysis. Women with only non-solid cancer or non-malignant tumours specified as benign tumours, non-melanoma skin cancer, hematologic cancer, desmoplasia and neoplasms of uncertain behaviour were excluded from the analysis. Lastly, women with a cancer diagnosis within the first 6 months after baseline were removed to reduce the risk of reverse causality (Fig. 1). In the case of a woman having more than one solid cancer diagnosis at least 6 months after baseline, only the first incident of cancer was included in the analysis.

### Statistical analysis

All continuous variables were normally distributed. Continuous and categorical baseline characteristics of women with and without a cancer diagnosis were compared using a t-test and a chi-squared test, respectively (Table 1). A Cox regression model was applied in both a univariable and multivariable regression fashion to estimate overall cancer risk (Table 2). In the multivariable analysis, the covariates selected for the final model was based on a directed acyclic graph (DAG) (Figure S1). The following covariates were included: BMI [Quartiles (<23.19; 23.19 ≤ 25.72; 25.72 ≤ 28.68; >28.68)]; Recreational walking (≥5 times/week, min. 10 minutes); Exercise habits (≥1 time/week); Level of education (school (9 years)/high school (12 years)/university (17 years)); Smoking habits (never/former/current); Childbirth (≥1 child); Alcohol consumption (≥7 units/week) and Central obesity [Quartiles (<0.71; 0.71 ≤ 0.86; 0.86 ≤ 1.02; >1.02)].

**Table 1:** Baseline characteristics for the studied women.

Characteristics	Total cohort (*n* = *4,679*)	Women with a cancer diagnosis (*n* = 809)	Non-cases*(*n* = *3,870*)	*P*-val **
Age at baseline (years)				
Mean ± SD (n)	70.7 ± 6.5 (4679)	71.5 ± 6.1 (809)	70.5 ± 6.6 (3870)	<0.0001
BMI (kg/m^2^)				
Mean ± SD (n)	26.2 ± 4.2 (4501)	26.3 ± 4.3 (773)	26.1 ± 4.2 (3728)	0.32
Q1, n/total (%)	1125/4501 (25.0)	188/773 (24.3)	938/3728 (25.2)	0.79
Q2, n/total (%)	1125/4501 (25.0)	192/773 (24.8)	933/3728 (25.0)	
Q3, n/total (%)	1125/4501 (25.0)	189/773 (24.5)	936/3728 (25.1)	
Q4, n/total (%)	1126/4501 (25.0)	204/773 (26.4)	921/3728 (24.7)	
Recreational walking (min. 10 minutes)				
≥5 times/week, yes/no	3356/1315	559/249	2797/1066	0.07
(total n) (%yes)	(72) (4671)	(69) (808)	(72) (3863)	
Exercise habits				
≥1 time/week, yes/no	3208/1463	539/269	2669/1194	0.20
(%yes) (total n)	(69)(4671)	(67)(808)	(69)(3863)	
Education				
School (9 years) n/total (%)	3357/4671 (71.9)	591/809 (73.1)	2766/3862 (71.6)	0.40
high school (12 years) n/total (%)	991/4671 (21.2)	158/809 (19.5)	833/3862 (21.6)	
University (17 years) n/total (%)	323/4671 (6.9)	60/809 (7.4)	263/3862 (6.8)	
Smoking				
Never, n/total (%)	2221/4671 (47.)	204/809 (25.2)	1888/3862 (48.9)	0.003
Former, n/total (%)	1395/4671 (29.9)	272/809 (33.6)	1123/3862 (29.1)	
Current, n/total (%)	1055/4671 (22.6)	333/809 (41.2)	851/3862 (22.0)	
Childbirth				
≥1 child, yes/no	4168/500	706/102	3462/398	0.06
(%yes) (total n)	(89) (4668)	(87) (808)	(90) (3860)	
Alcohol				
≥7 units/week, yes/no	1515/3127	275/530	1240/2597	0.33
(%yes) (total n)	(33) (4642)	(34) (805)	(32) (3837)	
Central Obesity				
Mean ± SD (n)	0.88 ± 0.23	0.91 ± 0.24	0.87 ± 0.23	0.0003
Q1 (n/total, %)	1059/4235 (25.0)	154/719 (21.4)	905/3516 (25.7)	0.005
Q2 (n/total, %)	1059/4235 (25.0)	167/719 (23.2)	892/3516 (25.4)	
Q3 (n/total, %)	1059/4235 (25.0)	186/719 (25.9)	873/3516 (24.8)	
Q4 (n/total, %)	1058/4235 (25.0)	212/719 (29.5)	846/3516 (24.1)	

*Women with no history of cancer.

**Women with a cancer diagnosis vs. non-cases. t-test for continuous data and chi-squared test for categorical data.

**Table 2:** Univariable and multiple Cox regression analyzing women diagnosed with cancer vs. non-cases*.

Factors	Univariable analysis		Multivariable regression analysis		n, cases
	HR (95% CI)	*P*-value	HR (95% CI)	*P*-value	
BMI					
Q1	(ref)	—	(ref)	—	1067, 181
Q2	0.95 (0.77–1.17)	0.63	0.90 (0.73–1.12)	0.34	1062, 176
Q3	0.99 (0.81–1.22)	0.95	0.91 (0.73–1.14)	0.42	1043, 179
Q4	0.97 (0.79–1.20)	0.81	0.88 (0.70–1.10)	0.26	1029, 177
Recreational walking					
(<5 times/week)	(ref)	—	(ref)	—	1153, 210
(≥5 times/week)	0.87 (0.74–1.02)	0.09	0.89 (0.76–1.05)	0.18	3048, 503
Exercise habits					
(<1 time/week)	(ref)	—	(ref)	—	1268, 229
(≥1 time/week)	0.89 (0.76–1.04)	0.15	0.93 (0.79–1.10)	0.40	2449, 484
Education					
School (9 years)	(ref)	—	(ref)	—	3007, 517
High school (12 years)	0.95 (0.79–1.14)	0.57	0.94 (0.78–1.13)	0.51	899, 143
University (17 years)	1.01 (0.76–1.34)	0.97	0.96 (0.72–1.28)	0.77	295, 53
Smoking					
Never	(ref)	—			1994, 294
Former	1.34 (1.13–1.59)	<0.001	1.29 (1.09–1.53)	0.003	1270, 246
Current	1.45 (1.20–1.76)	<0.001	1.39 (1.15–1.69)	<0.001	937, 173
Childbirth					
(<1 child)	(ref)	—	(ref)	—	448, 88
(≥1 child)	0.87 (0.70–1.10)	0.25	0.87 (0.70–1.10)	0.24	3128, 625
Alcohol					
(<7 units/week)	(ref)	—	(ref)	—	2815, 470
(≥7 units/week)	1.09 (0.93–1.28)	0.27	1.05 (0.89–1.23)	0.56	1386, 243
Central Obesity					
Q1	(ref)	—	(ref)	—	1053, 151
Q2	1.10 (0.88–1.37)	0.40	1.14 (0.90–1.43)	0.27	1049, 167
Q3	1.21 (0.97–1.50)	0.09	1.26 (1.00–1.58)	0.05	1048, 183
Q4	1.45 (1.17–1.79)	<0.001	1.50 (1.20–1.88)	<0.001	1051, 212

*Women with no history of cancer.

Univariable and multivariable adjusted analysis was conducted on the complete dataset: n = 4201, cases = 713.

Abbreviations: BMI, Body Mass Index.

The time-scale of the model was age. Participants entered the analysis with their baseline age and exited with their age when they first got a cancer diagnosis at least 6 months after baseline. Non-cases exited the analysis with their censoring-age being the age at the end of the study (December 2012), the age when leaving Denmark or the age at death, whichever came first. Age was used as time-scale in the Cox analyses to ensure that the estimation was based on comparisons of individuals of the same age to prevent confounding by age^19^.

Multivariable Cox regression models stratified by smoking habits were done by classifying the participants into three groups: never, former and current smokers. To test for trends in the categorical obesity variables, study participants were assigned with the median value within their quartile and the resulting variable was fitted as a continuous variable in the regression models.

Variance inflation factor (VIF) values were estimated for each regression model. None of the VIF values were larger than 3 indicating no existence of collinearity. All statistical analyses were performed using R (V.3.3.2) and the survival package (V.2.40.1). Level of statistical significance was 0.05.

### Ethics statement

The PERF study protocol was approved by the Copenhagen County Scientific Ethics Committee (jr: KA 99070 gm). All participants in the present analysis provided written informed consent. All methods were carried out in accordance with the approved guidelines and regulations.

## Results

### Cohort characteristics

Of the 4,679 women who were DXA-scanned in the PERF I study, 809 were diagnosed with cancer at least 6 months after baseline and 3870 women had no history of cancer (non-cases) (Fig. 1).

Table 1 summarizes the baseline characteristics of the cohort. On average, the women were 71 ± 6.5 years old (range: 49–89 years) with a BMI of 26.2 ± 4.2. Age at baseline, smoking habits and central obesity were significantly different between women with a cancer diagnosis and non-cases. None of the other variables differed significantly between the two groups. A correlation analysis showed that BMI and DXA-derived central obesity measurements were weakly correlated (R ≤ 0.35).

Previous history of disease for the women in the total cohort was distributed in the following way: 23% had a history of diseases of the circulatory system, 11% had a history of diseases of the respiratory system, 21% had a history of diseases of the digestive system and 25% had a history of diseases of the musculoskeletal system.

### Obesity and overall cancer risk

To determine if body fat distribution was associated with cancer risk, both univariable and multivariable Cox regression analyses were conducted (Table 2).

In the univariable Cox regression, the DXA-derived measure of central obesity showed strong positive significant associations with overall cancer risk, whereas BMI was not associated significantly with cancer risk (Table 2). In the multivariable Cox regression analysis the fourth quartile of central obesity was independently associated with increased overall cancer risk compared with the first quartile after adjustment for covariates age at baseline, BMI, smoking habits, education, recreational walking, exercise habits, childbirth and alcohol consumption (HR: 1.50, CI: 1.20-1.88) (Table 2). A test for trend over the quartiles of central obesity was also significant (p for trend: p < 0.001). BMI was not significantly associated with overall cancer risk, and a test for trend over the quartiles was likewise non-significant (p for trend: p = 0.56).

### Obesity and site-specific cancer risk

To determine whether the results were applicable to all types of cancer, five multivariable Cox regression analyses with different site-specific cancers were performed. The cancer diagnoses were distributed between the five site-specific categories in the following way: GI: n = 223; respiratory: n = 122; FGO: n = 108; breast: n = 185; other: n = 171.

For respiratory cancers, the results showed that the fourth quartile of central obesity was significantly associated with increased cancer risk when compared to the first quartile (HR: 2.01, CI: 1.17-3.47). For BMI both the third and fourth quartiles of BMI were significantly negatively associated with cancer risk compared to the first quartile (Table 3). A test for trend over the quartiles was significant for BMI and non-significant for central obesity (p for trend; BMI: p = 0.006, central obesity: p = 0.12).

**Table 3:** Multivariable Cox regression analysis*: Women diagnosed with different cancers types vs. non-cases**.

Factors	Respiratory Cancers	GI Cancers	Breast Cancer	FGO Cancers	Other Cancers
	HR (95% CI); *P*-value; [n, cases]	HR (95% CI); *P*-value; [n, cases]	HR (95% CI); *P*-value; [n, cases]	HR (95% CI); *P*-value; [n, cases]	HR (95% CI); *P*-value; [n, cases]
BMI					
Q1	(ref); [44,930]	(ref); [47,933]	(ref); [33,919]	(ref); [22,909]	(ref); [35,921]
Q2	0.52 (0.30–0.88); 0.02; [908, 22]	0.88 (0.58–1.34); 0.55; [933, 47]	0.98 (0.60–1.61); 0.93; [920, 34]	0.82 (0.45–1.50); 0.53; [910, 24]	1.24 (0.79–1.95); 0.35; [935, 49]
Q3	0.54 (0.31–0.93); 0.03; [887, 23]	1.23 (0.82–1.84); 0.31; [930, 66]	1.27 (0.78–2.08); 0.33; [906, 42]	0.59 (0.31–1.12); 0.11; [883, 19]	0.73 (0.44–1.24); 0.25; [893, 29]
Q4	0.39 (0.21–0.73); 0.003; [869, 17]	0.77 (0.49–1.22); 0.26; [894, 42]	1.63 (1.00–2.64); 0.05; [905, 53]	0.83 (0.45–1.53); 0.55; [882, 30]	0.84 (0.50–1.40); 0.49; [887, 35]
Recreational walking					
(<5 times/week)	(ref); [36,979]	(ref); [59,1002]	(ref); [44,987]	(ref); [26,969]	(ref); [45,988]
(≥5 times/week)	0.77 (0.51–1.17); 0.22; [2615, 70]	0.92 (0.68–1.26); 0.61; [2688, 143]	0.96 (0.68–1.37); 0.82; [2663, 118]	1.00 (0.63–1.59); 1.00; [2614, 69]	0.81 (0.57–1.17); 0.26; [2648, 103]
Exercise habits					
(<1 time/week)	(ref); [43,1082]	(ref); [62,1001]	(ref); [41,1080]	(ref); [35,1074]	(ref); [48,1087]
(≥1 time/week)	0.74 (0.49–1.10); 0.13; [2512, 63]	1.00 (0.73–1.35); 0.98; [2589, 140]	1.27 (0.88–1.82); 0.20; [2570, 121]	0.68 (0.44–1.04); 0.08; [2509, 60]	0.88 (0.62–1.26); 0.49; [2549, 100]
Education					
School (9 yrs)	(ref); [80,2570]	(ref); [141,2631]	(ref); [116,2606]	(ref); [74,2564]	(ref) [106,2596]
High school (12 yrs)	0.91 (0.56–1.49); 0.72; [777, 21]	1.02 (0.72–1.44); 0.92; [798, 42]	1.04 (0.71–1.52); 0.84; [792, 36]	0.68 (0.39–1.19); 0.18; [771, 15]	0.90 (0.59–1.36, 0.61) [785, 29]
University (17 yrs)	0.65 (0.26–1.62); 0.35; [247, 5]	1.24 (0.76–2.03); 0.38; [261, 19]	0.84 (0.44–1.61); 0.60; [252, 10]	0.78 (0.34–1.82); 0.57; [248, 6]	1.10 (0.61–1.97, 0.76) [255, 13]
Smoking					
Never	(ref); [9,1709]	(ref); [83,1783]	(ref); [78,1778]	(ref); [51,1751]	(ref); [73,1777]
Former	6.30 (2.90–13.66); <0.0001; [1057, 33]	1.41 (1.03–1.93); 0.03; [1099, 75]	1.07 (0.75–1.52); 0.72; [1077, 53]	1.04 (0.67–1.61); 0.86; [1058, 34]	1.13 (0.79–1.62); 0.51; [1075, 51]
Current	15.28 (7.26–32.13; <0.0001; [828, 64]	1.37 (0.94–1.99); 0.10; [808, 44]	1.03 (0.67–1.58); 0.89; [795, 31]	0.45 (0.23–0.89); 0.02; [774, 10]	0.79 (0.50–1.27); 0.33; [788, 24]
Childbirth					
(<1 child)	(ref); [13,373]	(ref); [24,384]	(ref); [18,378]	(ref); [10,370]	(ref); [23,383]
(≥1 child)	0.86 (0.48–1.55); 0.62; [3221, 93]	0.91 (0.59–1.41); 0.68; [3306, 178]	0.91 (0.56–1.50); 0.72; [3272, 144]	0.96 (0.50–1.86); 0.90; [3213, 85]	0.67 (0.42–1.05); 0.08; [3253, 125]
Alcohol					
(<7 units/week)	(ref); [67,2412]	(ref); [133,2478]	(ref); [101,2446]	(ref); [67,2412]	(ref); [102,2447]
(≥7 units/week)	1.03 (0.69–1.54); 0.89; [1182, 39]	1.04 (0.77–1.41); 0.78; [1212, 69]	1.33 (0.96–1.84); 0.09; [1204, 61]	0.92 (0.58–1.44); 0.70; [1171, 28]	0.92 (0.65–1.32); 0.65; [1189, 46]
Central Obesity					
Q1	(ref); [28,930]	(ref); [38,940]	(ref); [35,937]	(ref); [16,918]	(ref); [34,936]
Q2	1.02 (0.57–1.85); 0.94; [902, 20]	1.36 (0.88–2.11); 0.16; [933, 51]	1.04 (0.64–1.66); 0.89; [922, 40]	1.24 (0.63–2.45); 0.54; [902, 20]	1.08 (0.66–1.76); 0.76; [918, 36]
Q3	1.07 (0.58–1.97); 0.82; [886, 21]	1.56 (1.01–2.41); 0.04; [923, 58]	0.96 (0.59-1.57); 0.88; [903, 38]	1.93 (1.01–3.70); 0.05; [895, 30]	1.16 (0.71–1.91); 0.55; [901, 36]
Q4	2.01 (1.17–3.47), 0.01; [876, 37]	1.55 (0.99–2.41); 0.05; [894, 55]	1.26 (0.79–2.03); 0.33; [888, 49]	1.95 (1.00–3.78); 0.05; [868, 29]	1.41 (0.87–2.31); 0.17; [881, 42]

*Analysis was conducted on complete datasets: Respiratory cancers, n = 3594, cases = 106, GI cancers, n = 3690, cases = 202, Breast cancer, n = 3650, cases = 162, FGO cancers, n = 3583, cases = 95, Other cancer, n = 3636, cases = 148.

**Women with no history of cancer.

Abbreviations: GI, Gastrointestinal; FGO, Female Genital Organs; BMI, Body Mass Index.

For both GI and FGO cancers the analyses showed a significantly increased risk of cancer when comparing the first and third quartile of central obesity (GI: [HR: 1.56, CI: 0.04]; FGO: [HR: 1.93, CI: 1.01-3.70]). Only for FGO cancers, the same association was observed when comparing the fourth quartile of central obesity with the first quartile (HR: 1.95, CI: 1.00-3.78).

In contrast, BMI was non-significant in both analyses (Table 3). Trend analyses showed that increased central obesity was positively associated with risk of GI and FGO cancers (p for trend: GI: p = 0.033, FGO: p = 0.039). There was no significant trend between increased BMI and risk of GI and FGO cancers (p for trend: GI: p = 0.91, FGO: p = 0.28).

For breast cancer, central obesity was not significantly associated with cancer risk, but here the fourth quartile of BMI was significantly associated with increased breast cancer risk when compared to the first quartile (HR: 1.63, CI: 1.00-2.64). In a test for trend, BMI was also significant in the analysis of breast cancer (p for trend = 0.01).

Neither BMI or central obesity was associated with an increased risk of other cancers (Table 3).

### Central obesity and smoking habits

To determine whether central obesity was independently associated with cancer risk regardless of smoking habits, the women were stratified into three groups; never smokers (n = 2,229), former smokers (n = 1,403) and current smokers (n = 1,063). The prevalence of a solid cancer diagnosis at least 6 months after baseline within the three groups was distributed in the following way; never smokers: 18% (n = 333), former smokers: 24% (n = 272) and current smokers: 24% (n = 204).

A multivariable Cox regression analysis adjusted for covariates revealed that the fourth quartile of central obesity was significantly associated with increased risk for overall cancer diagnosis in current smokers when compared to the first quartile of central obesity (HR: 1.93, CI: 1.25-2.99). In former smokers, the second, third and fourth quartiles of central obesity were all significantly associated with increased overall cancer risk (Q1vs.Q2: [HR: 1.79, CI: 1.16-2.75], Q1vs.Q3: [HR: 1.89, CI: 1.23-2.93], Q1vs.Q4: [HR: 1.90, CI: 1.23-2.94]) (Table 4). A test for trend over the quartiles of central obesity was significant both in the group of former and current smokers. In former smokers, the second quartile of BMI was significantly associated with increased overall cancer risk when compared to the first quartile of BMI. In never smokers, neither BMI or central obesity were significantly associated with overall cancer risk. When stratifying for both smoking habits and site-specific cancer the sample-size became too small for a meaningful analysis.

**Table 4:** Multivariable Cox regression analysis*. Women diagnosed with cancer vs. non-cases** stratified by smoking status.

Factors	Never smokers	Former smokers	Current smokers
	HR (95% CI); *P*-value; [n, cases]	HR (95% CI); *P*-value; [n, cases]	HR (95% CI); *P*-value; [n, cases]
BMI			
Q1	(ref) [58,438]	(ref) [59,289]	(ref) [64,340]
Q2	1.15 (0.81–1.63); 0.43; [521, 79]	0.66 (0.45–0.97); 0.04; [301, 51]	0.89 (0.60–1.33); 0.58; [240, 46]
Q3	1.15 (0.80–1.65); 0.45; [508, 78]	0.74 (0.50–1.09); 0.13; [326, 61]	0.86 (0.57–1.31); 0.49; [209, 40]
Q4	1.11 (0.77–1.61); 0.57; [527, 79]	0.80 (0.55–1.16); 0.24; [354, 75]	0.61 (0.36–1.01); 0.05; [148, 23]
Recreational walking			
(<5 times/week)	(ref) [91,517]	(ref) [66,357]	(ref) [53,279]
(≥5 times/week)	0.75 (0.58–0.96); 0.02; [1477, 203]	1.07 (0.81–1.43); 0.63; [913, 180]	0.96 (0.69–1.33); 0.79; [658, 120]
Exercise habits	(ref)		
(<1 time/week)	(ref) [83,541]	(ref) [74,359]	(ref) [72,368]
(≥1 time/week)	1.05 (0.81–1.36); 0.72; [1453, 211]	0.91 (0.69–1.20); 0.49; [911, 172]	0.85 (0.62–1.15); 0.29; [569, 101]
Education			
School (9 years)	(ref) [207,1409]	(ref) [177,892]	(ref) [133,706]
High school (12 years)	1.00 (0.75–1.33); 1.00; [439, 62]	0.93 (0.68–1.28); 0.66; [274, 52]	0.83 (0.55–1.25); 0.37; [186, 29]
University (17 years)	1.10 (0.72–1.68); 0.66; [146, 25]	0.75 (0.45–1.24); 0.26; [104, 17]	1.11 (0.59–2.08); 0.75; [45, 11]
Childbirth			
(<1 child)	(ref) [30,191]	(ref) [31,147]	(ref) [27,110]
(≥1 child)	0.98 (0.67–1.43); 0.91; [1803, 264]	0.89 (0.61–1.30); 0.55 [1123, 215]	0.74 (0.49–1.12); 0.15; [827, 146]
Alcohol			
(≥7 units/week)	(ref) [209,1426]	(ref) [149,785]	(ref) [112,604]
(≥7 units/week)	1.04 (0.80–1.34); 0.79; [568, 85]	1.10 (0.85–1.43); 0.47 [485, 97]	0.97 (0.71–1.34); 0.86; [333, 61]
Central obesity			
Q1	(ref) [75,508]	(ref) [35,272]	(ref) [41,273]
Q2	0.79 (0.56–1.12); 0.19; [533, 66]	1.79 (1.16–2.75); 0.008; [309, 64]	1.25 (0.79–1.98); 0.34; [207, 37]
Q3	0.91 (0.65–1.28); 0.60; [508, 74]	1.89 (1.23–2.93); 0.004; [324, 70]	1.38 (0.87–2.19); 0.17; [216, 39]
Q4	1.16 (0.82–1.63); 0.40; [445, 79]	1.90 (1.23–2.94); 0.004; [365, 77]	1.93 (1.25–2.99); 0.003; [241, 56]

*Analysis was conducted on the complete datasets: Never smokers, n = 1994, cases = 294; Former smoker, n = 1270, cases = 246; Current smokers, n = 937, cases = 173.

**Women with no history of cancer.

Abbreviations: BMI, Body Mass Index.

## Discussion

In this prospective study including 4,679 postmenopausal women, we found that (i) high central obesity was independently associated with increased risk of overall cancers, (ii) high central obese women had significantly increased risk of respiratory, GI and FGO cancer when compared to low central women and (iii) when stratifying the analysis by smoking habits, high central obesity was significantly associated with cancer risk in former and current smokers.

These findings extend our understanding of the link between obesity and cancer and provide strong support for a positive association between central obesity and risk of some cancers in postmenopausal women. Furthermore, our findings underline the importance of including measures of body fat distribution in risk analyses, as obesity derived from BMI and body fat distribution describe two different obesity-types that have a different impact on risk.

Several studies have investigated the relationship between different cancer subtypes and anthropometric measures of obesity^5,20^. In alignment with our results, Wang *et al*. found a significant association between WC and increased risk of colorectal cancer in a cohort of 87,261 women^21^. For lung cancer, previous studies have shown an increased BMI to be a protective factor^22^. Reeves *et al*. found a 34% decreased risk of lung cancer for every 10 unit increase in BMI in a cohort of 1.2 million elderly women^23^. These results are consistent with our results which showed that an elevated BMI was inversely associated with respiratory cancer risk. In contrast, we found a significant positive association between high central obesity and respiratory cancers after adjusting for BMI and other cofounders. This suggests that while a high BMI is protective for respiratory cancer, high central obesity might be a risk factor. These results substantiate a recent meta-analysis, which concluded that central obesity, measured by WC and WHR, may play a role in the development of lung cancer^24^.

The biological mechanism behind the contradictory association between central obesity and general obesity to lung cancer remains poorly understood. Possible explanations could involve hyperinsulinemia and increased unbound estrogens, both of which have a stronger correlation to central obesity compared to general obesity^24,25^. Other studies have also suggested that reduced oxidative DNA damage, which is inversely correlated to BMI, could explain the protective effect of a high BMI in lung cancer^26,27^. Nonetheless, further research is warranted to understand the associations between central obesity and general obesity to lung cancer.

The link between obesity and hormone-associated cancers, such as breast, endometrial and ovarian cancer, is well established in postmenopausal women and thought to be mediated by increased concentrations of circulating sex hormones^23,28^. In the Million Women Study, a 10 unit increase in BMI was associated with a four times higher likelihood for developing endometrial cancer^23^, which is in line with our results where central obesity is significantly associated with FGO cancer. Furthermore, in a study of 10,960 elderly women, Rohan *et al*. showed a strong positive association between both BMI and ratio of trunk fat mass to average of legs fat mass and breast cancer^28^. In this study, we also found a significant association between BMI and breast cancer, but central obesity did not show any significant association to breast cancer risk, though a similar trend for central obesity was observed. An explanation for the conflicting results might be the lower power in this study or that we in this study include both BMI and central obesity in the analysis whereas Rohan *et al*. perform separate analyses for the obesity measures.

Postmenopausal women tend to experience a re-distribution of body fat from a gynoid to an android pattern which has been linked to the metabolic syndrome and increased risk of cardiovascular diseases^28^. In line with this, we have previously seen, in a PERF subpopulation, that localization of fat mass was more important for atherosclerosis than general obesity^29^. Level of physical activity may also influence the accumulation of fat. In a recent study including 10,976 individuals from the general population^30^, cardiorespiratory fitness was found to be inversely associated with both central obesity and low-grade inflammation independent of BMI. Moreover, physical inactivity is an independent cause of the accumulation of visceral fat^31^ and regular exercise decreases central obesity and exerts anti-inflammatory effects^32^. Thus, high central obesity may reflect low fitness and low-grade inflammation, which are both associated with an increased risk of cancer^33^.

Smoking is also known to affect the body fat distribution resulting in smokers having a higher waist circumference compared to non-smoker^34,35^. Furthermore, an association between lung cancer and BMI in former smokers has previously been shown^36^, though others have provided evidence that obesity may act as a protective factor against lung cancer^37^. In this study we found that high central obesity was significantly associated with increased risk of overall cancer in former and current smokers, but not in never smokers. We also found that the second quartile of BMI was inversely associated with overall cancer risk in former smokers. This substantiates the claim of stratification by smoking habits when assessing obesity and cancer risk^13^, and suggests that in former and current smokers general and central obesity reflect different risk.

As a whole, the results of this paper support the growing evidence that the distribution of body fat after menopause is as important as the fat mass itself when studying the risk of obesity-related diseases^28^. This article adds to the knowledge about age-related diseases and postmenopausal obesity. Understanding the complex biology underlying the association between central obesity and menopause could, therefore, serve, not only as prevention strategies for cardiovascular diseases but also as cancer prevention strategies.

Limitations of this study include the number of cancer diagnoses, which was low when both sub-dividing for site-specific cancer and stratifying by smoking habits. Due to the small sample size, it was also impossible to subdivide the cancer-types even further by individual cancer sites. The data is restricted to postmenopausal Danish Caucasian women and may therefore not be generalizable to the general world population. The choice of variables to include in the analyses was restricted to the data present in the PERF study. The often-used measures WHR and WC were not recorded at baseline, why it was impossible to compare these measures with the central obesity measure from the DXA-scanning. Lastly, the study design does not allow an assessment of the causal role of central obesity in relation to cancer risk. The strength of this study includes the prospective design, the long follow up period and the access to the Danish health registries. Furthermore, the PERF cohort represents a limited number of cohorts where postmenopausal women are evaluated with DXA-scans^38^.

In conclusion, our study supports several relevant hypotheses. Firstly, we show that central obesity is independently associated with an increased risk of overall cancers. Secondly, we show that women with high central obesity have more than 50% increased risk of respiratory, GI and FGO cancers respectively compared to women with low central obesity. Lastly when stratifying the analysis by smoking habits high central obesity was significantly associated with cancer risk in former and current smokers. These results suggest that central obesity is at least partially independent of BMI and that different types of obesity have a diverse impact on health. This underlines that health strategies on keeping a healthy weight should be supplemented by recommendation on preventing central obesity, especially for women facing the menopause transition.

## Supplementary information

Supplementary figure 1

## Data Availability

The original data of the Prospective Epidemiological Risk Factor study and the linkage data from various health registries are currently stored in Nordic Bioscience. Access to this database will be granted, on condition that researchers have appropriate ethical permission and sign the appropriate Material Transfer Agreement form.
