# Supplementary material for: Cancer risk in relation to body fat distribution, evaluated by DXA-scans, in postmenopausal women – the Prospective Epidemiological Risk Factor (PERF) study
Source: Sci Rep. 2019 Mar 29;9:5379. doi: 10.1038/s41598-019-41550-1 (PMC6440966; doi:10.1038/s41598-019-41550-1)
Supplement: Supplementary file 1 — Supplementary figure 1 [file 41598_2019_41550_MOESM1_ESM.pdf]

Cancer risk in relation to body fat distribution, evaluated by DXA-scans, in postmenopausal women – the Prospective Epidemiological Risk Factor (PERF) study, Line Mærsk Staunstrup, Henning Bay Nielsen, Bente Klarlund Pedersen, Morten Karsdal, Joseph Patrick Michele Blair, Jesper Frank Christensen, Cecilie Liv Bager.

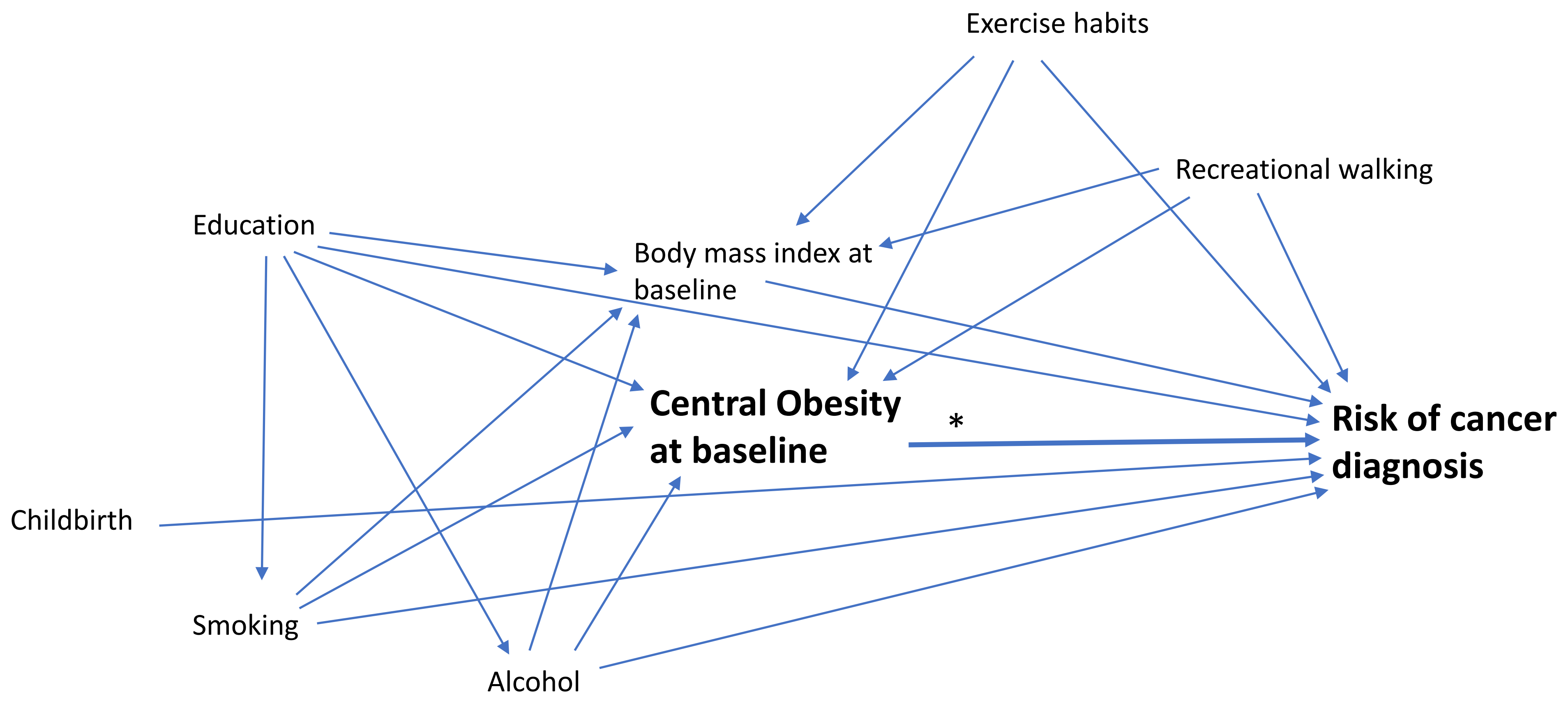

Figure S1: Directed acyclic graph (DAG) illustrating assumed causal structure underlying our adjusted Cox regression analysis.

**Causal path of interest*
